# SENESCENCE SECRETOME: BIOMARKERS OF BIOLOGICAL AGING AND POSTOPERATIVE OUTCOMES

**DOI:** 10.1093/geroni/igz038.370

**Published:** 2019-11-08

**Authors:** Marissa Schafer, Xu Zhang, Amanika Kumar, Thomas White, Elizabeth Atkinson, Michelle Burning, Nathan LeBrasseur

**Affiliations:** 1 Mayo Clinic, Rochester, Minnesota, United States; 2 Robert and Arlene Kogod Center on Aging, Mayo Clinic, Rochester, Minnesota, United States

## Abstract

Senescent cells drive age-related tissue dysfunction through their potent secretome, termed the senescence associated secretory phenotype (SASP). Circulating concentrations of SASP factors may reflect biological age and serve as clinically useful biomarkers of surgical risk and ultimately, surrogate endpoints in clinical trials. However, they remain largely uncharacterized. We tested associations between circulating concentrations of SASP proteins and biological age, as determined by the accumulation of age-related health deficits, and/or postoperative outcomes in a sample of residents in Olmstead County, MN, age 60-90 years (n = 115) and cohorts of older adults undergoing surgery for severe aortic stenosis (prospective; n = 97) or ovarian cancer (case-control; n = 36). Circulating concentrations of SASP factors were associated with biological age and adverse postoperative outcomes, including risk of any adverse event or rehospitalization within the year following surgery (aortic stenosis group) or admission to an intensive care unit within 30 days of hospital discharge (ovarian cancer group). Gradient boosting machine modeling revealed a panel of SASP factors that predicted adverse outcomes across both surgical groups better than biological age or chronological age and sex. This suggests that the circulating SASP is a robust indicator of age-related health status and may help guide clinical decision making. Furthermore, circulating SASP factors may be harnessed as a readily quantifiable biomarkers in senescence-targeting interventional human studies.

